# Apremilast, an oral phosphodiesterase 4 (PDE4) inhibitor: A novel treatment option for nurse practitioners treating patients with psoriatic disease

**DOI:** 10.1002/2327-6924.12428

**Published:** 2016-11-21

**Authors:** Melodie Young, Heather L. Roebuck

**Affiliations:** ^1^ Modern Dermatology–Aesthetics Center DallasDallasTexas; ^2^ Modern Research AssociatesDallasTexas; ^3^ RoebuckDERMWest BloomfieldMichigan

**Keywords:** Dermatology, psoriasis, medications, patient, treatment

## Abstract

**Background and purpose:**

Apremilast is an oral nonbiologic medication approved for the treatment of adult patients with active psoriatic arthritis and for patients with moderate to severe plaque psoriasis. This article summarizes the efficacy and safety of apremilast and provides characterization of the novel medication with clinical perspectives to successfully incorporate this therapy into practice for appropriate patients.

**Data sources:**

A review and synthesis of the results from the ESTEEM (Efficacy and Safety Trial Evaluating the Effects of Apremilast in Psoriasis) phase 3 clinical studies evaluating the efficacy, safety, and tolerability of apremilast for the treatment of moderate to severe plaque psoriasis was conducted.

**Conclusions:**

Results from the ESTEEM clinical trial program demonstrate that apremilast significantly reduces the severity of moderate to severe plaque psoriasis, has an acceptable safety profile, and is generally well tolerated.

**Implications for practice:**

The novel mechanism of action, convenience of oral administration, and acceptable side effect profile make this medication an attractive choice for clinicians treating patients with plaque psoriasis.

## Introduction

Psoriasis is a chronic, immune‐mediated, inflammatory disease characterized by distinctive, red, raised skin lesions with adherent silvery scales (Nestle, Kaplan, & Barker, 2009). This disorder affects approximately 1%–3% of the population worldwide (Parisi, Symmons, Griffiths, & Ashcroft, 2013). Psoriasis lesions most commonly appear on the elbows, knees, scalp, and trunk and may persist for months, for years, or throughout the patient's life (Johnson & Armstrong, 2013; Myers, Gottlieb, & Mease, 2006). These lesions can cause physical symptoms of itching, flaking, redness, and pain (Lebwohl et al., 2014; Martin et al., 2015). Visually apparent skin lesions and the associated discomfort caused by these plaques contribute to significant impairments in patients’ quality of life (Kimball, Jacobson, Weiss, Vreeland, & Wu, 2005; Weiss et al., 2002). Furthermore, psoriasis is associated with multiple physical and psychological comorbidities, and afflicted patients have a higher risk than the general population for conditions such as psoriatic arthritis, metabolic syndrome, and cardiovascular disease (Garshick & Kimball, 2015; Gottlieb & Dann, 2009).

Psoriasis is a lifelong recurrent disease that often requires ongoing treatment to reduce disease symptoms, improve quality of life, and maintain remission (Van Voorhees et al., 2016). Management strategy depends on the severity of disease and comorbidities, and should account for the specific needs of the patient (Blome et al., 2016). The present armamentarium, comprised of topical medications, phototherapy, conventional systemic medications, and biologic agents, offers excellent options for the treatment of psoriasis. Despite the availability of various treatments and strategies, results of recent surveys have demonstrated widespread nontreatment and undertreatment of patients with psoriasis, regardless of disease severity (Armstrong, Robertson, Wu, Schupp, & Lebwohl, 2013; Lebwohl, Kavanaugh, Armstrong, & Van Voorhees, 2016). Treatment dissatisfaction resulting in poor treatment adherence and discontinuation among patients with psoriasis hinders the achievement of optimal outcomes. Lack of clinical response, loss of therapeutic response over time, and safety and tolerability concerns are common reasons for discontinuation of conventional systemic and biologic therapies (Lebwohl et al., 2016; Levin, Gottlieb, & Au, 2014). Adherence improves when treatments are efficacious, protocols are easy to follow, the treatment or medication is convenient, and safety concerns about medications are minimal (Bewley & Page, 2011). The development of orally available small molecules that regulate the production of inflammatory mediators within the psoriasis signaling pathway may address unmet needs for patients who are intolerant of conventional nonbiologic therapies or who are not candidates for biologic treatments (Alwan & Nestle, 2015; Schafer, 2012).

### Introduction to apremilast

Apremilast is a small‐molecule (nonbiologic) oral phosphodiesterase 4 (PDE4) inhibitor that works intracellularly to regulate production of pro‐ and anti‐inflammatory mediators implicated in the pathogenesis of psoriasis and psoriatic arthritis (Schafer et al., 2010). It is an effective and well‐tolerated medication for the treatment of psoriasis. In 2014, apremilast was approved by the U.S. Food and Drug Administration for the treatment of adult patients with active psoriatic arthritis and for patients with moderate to severe plaque psoriasis who are candidates for phototherapy or systemic therapy (Celgene Corporation, 2015). Apremilast has since been approved in multiple countries, including the European Union, Canada, and Australia (Celgene, Inc., 2015; Celgene Pty Limited, 2015; European Medicines Agency, 2015). As the first and only selective PDE4 inhibitor approved for patients with these conditions, apremilast provides clinicians with a new therapeutic option for patients in need of alternative medications to manage the symptoms of psoriatic disease.

## Mechanism of action and review of pharmacodynamics of apremilast

**Figure 1:**
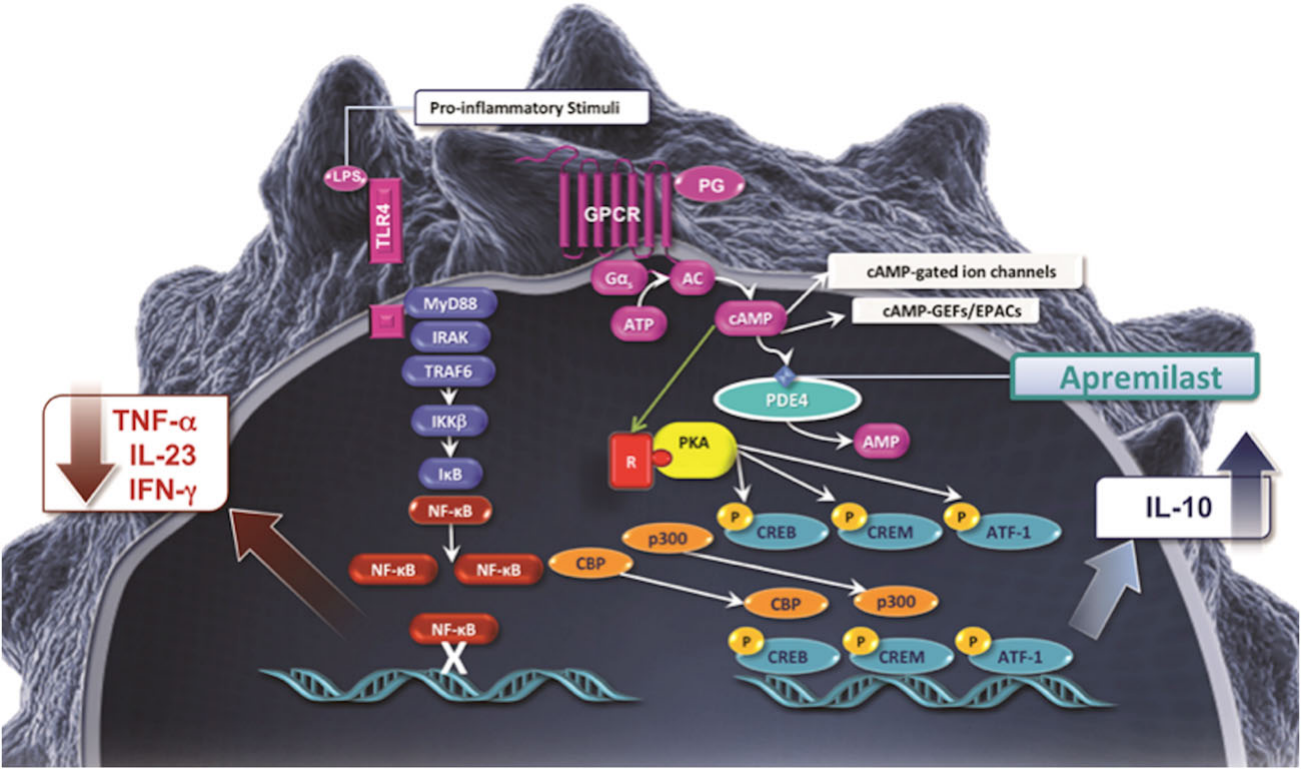
Mechanism of action of apremilast.

Apremilast works intracellularly to regulate the production of multiple inflammatory mediators by specifically inhibiting PDE4 (Figure 1; Man et al., 2009; Schafer, 2012). PDE4 degrades intracellular cyclic adenosine monophosphate (cAMP). As a secondary messenger within the cell, cAMP regulates inflammation by suppressing or inhibiting the expression of proinflammatory cytokines, such as tumor necrosis factor‐alpha (TNF‐α), interferon‐gamma (IFN‐γ), and interleukin 23 (IL‐23), and by promoting the release of anti‐inflammatory cytokines such as IL‐10 (Houslay, Schafer, & Zhang, 2005; Schafer et al., 2010). PDE4 inhibition increases cAMP, which in turn causes inflammation responses within T‐helper cell 1 (Th1), Th17, and type 1 IFN pathways to be downregulated. In addition, elevated cAMP modulates the production of IL‐10 (Schafer et al., 2014).

The effect of apremilast on inflammatory mediators was demonstrated in a phase 2 open‐label study by analyzing inflammatory markers found in lesional skin biopsies from patients receiving apremilast 20 mg twice daily (BID; Gottlieb et al., 2013). At weeks 4 and 12, significant reductions were observed in several inflammatory markers, including IL‐12/IL‐23p40 (week 4: *p* = .025; week 12: *p* = .039) and IL‐17A (week 4: *p* = .021; week 12: *p* = .031; Gottlieb et al., 2013). Levels of the anti‐inflammatory mediator IL‐10 were increased in patients who were classified as responders (patients who achieved ≥75% improvement in Psoriasis Area and Severity Index [PASI‐75]) but decreased in nonresponders (Gottlieb et al., 2013). Similar pharmacodynamic impacts of apremilast were found in a phase 3 psoriatic arthritis clinical trial substudy (Schafer, Chen, Fang, Wang, & Chopra, 2015). Plasma samples from 150 randomized patients were collected at weeks 4, 16, 24, and 40 and assessed for a broad array of inflammatory biomarkers. At 40 weeks, IL‐6, IL‐17, and IL‐23 showed significant inhibition in patients receiving apremilast 30 mg BID treatment, and IL‐10 had a significant increase from baseline levels (Schafer et al., 2015). Additionally, during the placebo‐controlled period (weeks 0‒24) in the apremilast treatment arms, reductions in multiple inflammatory biomarkers (TNF‐α, IL‐8, and macrophage inhibitory protein‐1β) were seen as early as week 4 in patients receiving apremilast compared with placebo (*p* ≤ .0527). These effects were consistent through week 24 (Schafer et al., 2015). These data indicate that apremilast may impact innate and Th1 inflammation in the early stage of treatment followed by regulation of components of the systemic Th17 immune response after continued treatment (Schafer et al., 2015).

The molecular mechanism whereby apremilast alters the pathophysiology of psoriatic disease is not fully understood. Psoriasis is driven by dysregulation of the cellular immune system, leading to overproduction of cytokines and chemokines released by the innate and adaptive immune systems (Lowes, Bowcock, & Krueger, 2007; Schafer, 2012). When PDE4 inhibitors such as apremilast are introduced into the cell, the resulting increase in cAMP levels in immune cells helps to decrease the inflammation that occurs in psoriasis and psoriatic arthritis (Schafer, 2012).

## Pharmacokinetics of apremilast

The mean half‐life (*t*_1/2_) for apremilast is 6–9 h, and the time to reach maximum concentration (*T*_max_) in serum is approximately 2.5 h (Celgene Corporation, 2015). Apremilast is initially metabolized via cytochrome P450 (CYP)‐mediated pathways and subsequently via glucuronidation and non–CYP‐mediated hydrolysis; the major metabolites are inactive (Hoffmann et al., 2011; Liu, Zhou, Wan, Wu, & Palmisano, 2014). As indicated in the U.S. product label, apremilast should not be coadministered with drugs that are strong CYP3A4 inducers (e.g., rifampin, phenobarbital, carbamazepine, phenytoin) because this may result in a decrease in efficacy of apremilast (Celgene Corporation, 2015). Apremilast is eliminated in urine (58% of dose) and feces (39% of dose), with <10% of the dose eliminated as unchanged apremilast molecule (Hoffmann et al., 2011).

Hepatic impairment does not affect apremilast pharmacokinetics, and no dosage adjustment is necessary for patients with hepatic insufficiency (Celgene Corporation, 2015). Apremilast exposure may be greater in patients with severe renal impairment; therefore, maintenance dose for patients with creatinine clearance < 30 mL/min (estimated by the Cockcroft–Gault equation) should be reduced to 30 mg once daily, and the initial dose titration for these patients should be modified such that patients follow the titration schedule shown in Table 1 for the morning doses and omit the evening doses (Celgene Corporation, 2015).

**Table 1:** Five‐day apremilast titration schedule

Day 1	Day 2		Day 3		Day 4		Day 5		Day 6 and thereafter	
AM	AM	PM	AM	PM	AM	PM	AM	PM	AM	PM
10 mg	10 mg	10 mg	10 mg	20 mg	20 mg	20 mg	20 mg	30 mg	30 mg	30 mg

*Note*. Dosage is titrated upward during the first 6 days to reduce the risk for gastrointestinal symptoms and to achieve the recommended dose of 30 mg twice daily (BID).

Metabolites for apremilast were detected in milk of lactating mice but have not been assessed in humans; caution is recommended when administering apremilast to nursing women (Celgene Corporation, 2015). Apremilast is pregnancy category C. Potential benefits may warrant use of the drug in pregnant women; however, there have been no adequate and well‐controlled studies in humans to detect adverse effects of apremilast on a fetus (Celgene Corporation, 2015).

## Overview of ESTEEM 1 and ESTEEM 2 phase 3 trials

Two phase 3 multicenter, multinational, randomized, double‐blind, placebo‐controlled trials ESTEEM 1 (NCT01194219) and ESTEEM 2 (NCT01232283) were conducted to demonstrate efficacy and safety of apremilast in plaque psoriasis (Papp et al., 2015; Paul et al., 2015). Both studies included patients aged 18 years or older with moderate to severe chronic plaque psoriasis defined as PASI score ≥ 12, body surface area (BSA) involvement of ≥10%, and static Physician Global Assessment (sPGA) score ≥3 (moderate or severe disease; Papp et al., 2015; Paul et al., 2015). Eligible patients were candidates for phototherapy and/or systemic therapy (conventional or biologic). Patients with a history of phototherapy or systemic therapy, including previous treatment failures, were permitted to enroll. Patients were excluded, however, if they used biologics within 12–24 weeks, other systemic agents or phototherapy within 4 weeks, topical antipsoriatic agents within 2 weeks, or if they had prolonged sun exposure or used other ultraviolet light sources (Papp et al., 2015; Paul et al., 2015). Additionally, patients were excluded if they had a history of other clinically significant disease or other major uncontrolled disease, or if they had active tuberculosis infection or history of incompletely treated tuberculosis (screening for latent tuberculosis infection was not required; Papp et al., 2015; Paul et al., 2015).

**Figure 2:**
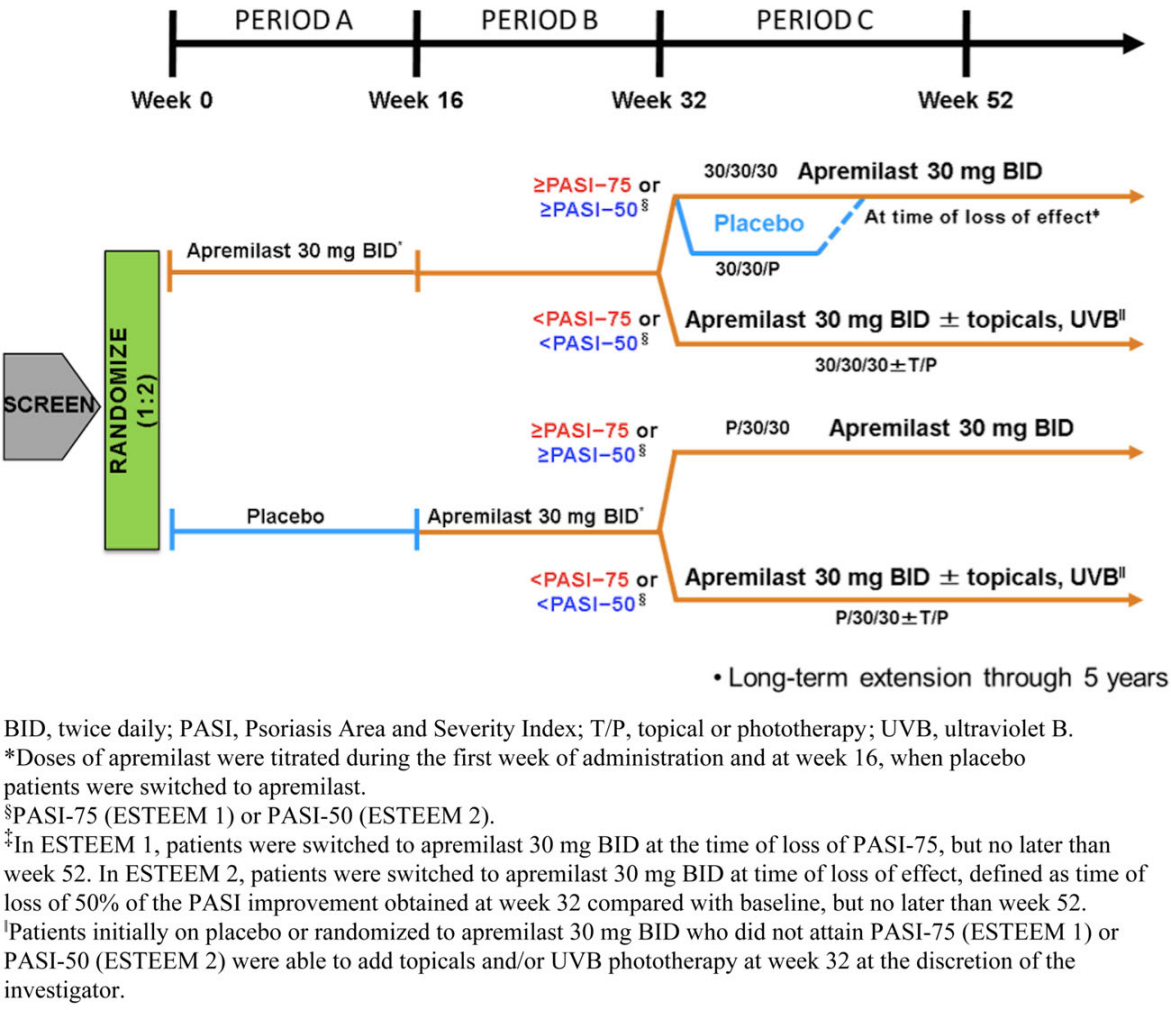
Study design for ESTEEM 1 and ESTEEM 2.

Both ESTEEM 1 and ESTEEM 2 consisted of three treatment periods designated as A, B, and C (Figure 2; Papp et al., 2015; Paul et al., 2015). Period A was a placebo‐controlled phase lasting from weeks 0 to 16. During this period, patients were randomized (2:1) to receive apremilast 30 mg or placebo, BID (Papp et al., 2015; Paul et al., 2015). A blinded dose‐titration pack was used for the first 6 days of treatment (Table 1). Period B was a maintenance phase lasting from weeks 16 to 32. All patients were treated with apremilast 30 mg BID during this time (Papp et al., 2015; Paul et al., 2015). As at baseline, a blinded dose‐titration regimen was used for the first 6 days of apremilast treatment. Period C consisted of a randomized treatment withdrawal phase lasting from weeks 32 to 52. During Period C, patients initially randomized to receive apremilast (Period A) who achieved a ≥75% reduction from baseline in PASI score (PASI‐75; ESTEEM 1) or a ≥50% reduction from baseline in PASI score (PASI‐50; ESTEEM 2) at week 32 were re‐randomized (1:1, blinded) to continue treatment with apremilast or switch to placebo (Papp et al., 2015; Paul et al., 2015). Patients re‐randomized to receive placebo who lost response in ESTEEM 1 (defined as loss of PASI‐75) or ESTEEM 2 (defined as loss of 50% of the improvement of PASI score obtained at week 32 relative to baseline) resumed treatment with apremilast 30 mg BID, without titration (Papp et al., 2015; Paul et al., 2015). During Period C, patients who had been randomized to the placebo group at baseline and were responders at week 32 were maintained on apremilast 30 mg BID alone until week 52. Patients who did not achieve PASI‐75 (ESTEEM 1) or PASI‐50 (ESTEEM 2) at week 32, regardless of initial treatment assignment, were continued on apremilast 30 mg BID, and topical therapies and/or ultraviolet B (UVB) phototherapy were added at the investigator's discretion (Papp et al., 2015; Paul et al., 2015). All patients were subsequently eligible to enter a long‐term open‐label extension study in which treatment with apremilast 30 mg BID was continued for up to four additional years.

### Clinical efficacy

**Figure 3:**
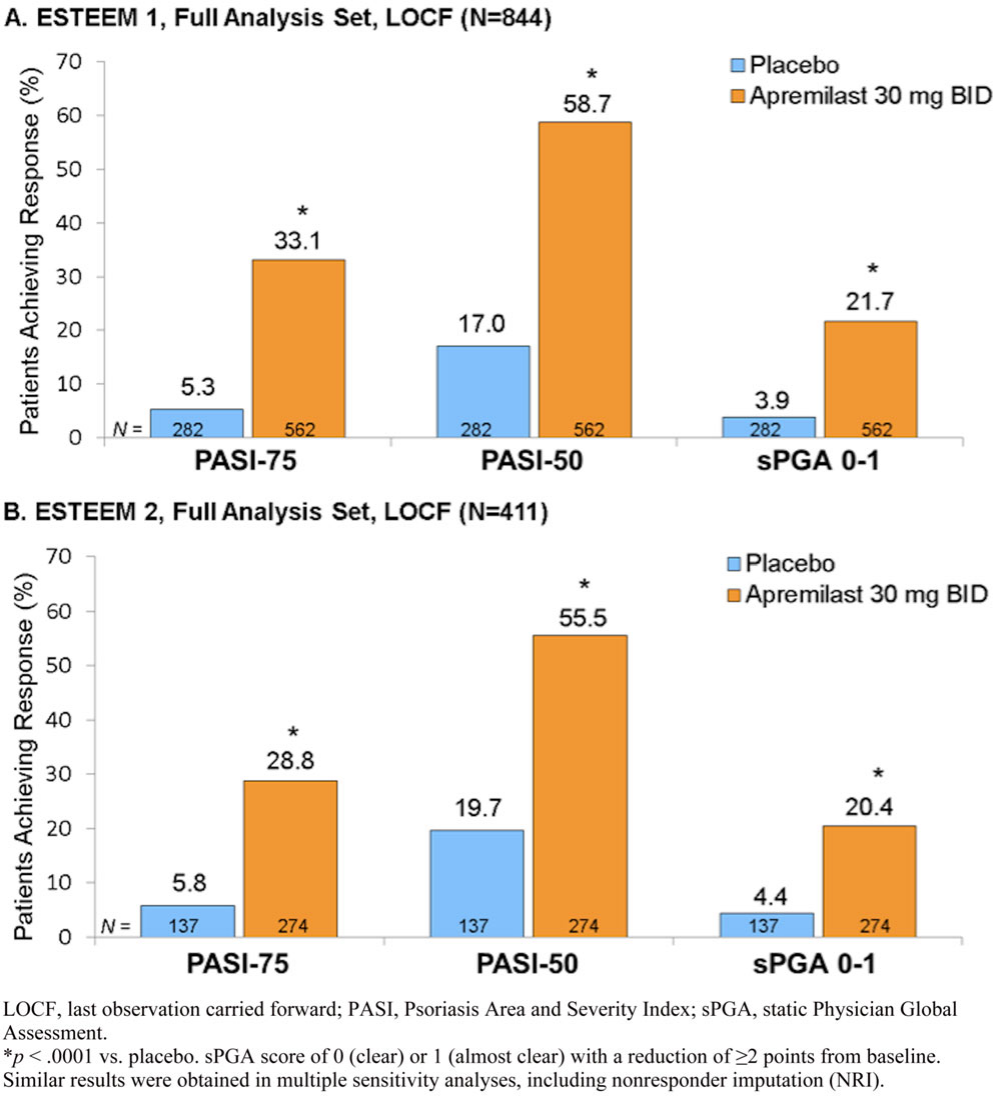
PASI‐75, PASI‐50, and sPGA response at week 16 for (A) ESTEEM 1 and (B) ESTEEM 2.

Baseline demographics and disease characteristics for the ESTEEM trials are provided in Table 2 (Reich, Papp et al., 2015). The primary end point was the proportion of patients who achieved PASI‐75 at 16 weeks (Figure 3; Papp et al., 2015; Paul et al., 2015). Significantly more patients receiving apremilast achieved a PASI‐75 response versus placebo in both ESTEEM 1 (33.1% vs. 5.3%; *p* < .0001) and ESTEEM 2 (28.8% vs. 5.8%; *p* < .0001; Papp et al., 2015; Paul et al., 2015). More than half of patients in both ESTEEM 1 and ESTEEM 2 receiving apremilast achieved a PASI‐50 response versus placebo (58.7% vs. 17.0%; and 55.5% vs. 19.7%; for ESTEEM 1 and ESTEEM 2, respectively, both *p* < .0001). Significantly more patients achieved an sPGA score of 0 (clear) or 1 (almost clear) with a ≥ 2‐point reduction from baseline compared with placebo at week 16 in both studies (*p* < .0001; Figure 3; Papp et al., 2015; Paul et al., 2015).

**Table 2:** ESTEEM 1 and ESTEEM 2 pooled baseline demographics and disease characteristics: Full analysis set

	Placebo *n* = 419	Apremilast (30 mg) BID *n* = 836
Age, mean, years (*SD*)	46.2 (12.9)	45.6 (13.1)
Male, *n* (%)	294 (70.2)	555 (66.4)
White, *n* (%)	378 (90.2)	757 (90.6)
Body mass index, mean, kg/m^2^ (*SD*)	31.1 (7.3)	31.1 (6.7)
Weight, mean, kg (*SD*)	92.7 (23.0)	92.6 (21.9)
Duration of plaque psoriasis, mean, years (*SD*)	18.7 (12.3)	19.2 (12.5)
PASI score (0–72), mean (*SD*)	19.6 (7.6)	18.8 (7.1)
PASI score > 20, *n* (%)	136 (32.5)	239 (28.6)
Body surface area, mean, % (*SD*)	26.1 (15.1)	24.8 (14.9)
Body surface area > 20%, *n* (%)	229 (54.7)	409 (48.9)
sPGA = 4 (severe), *n* (%)	138 (32.9)	236 (28.2)
Prior systemic therapy (conventional and/or biologics), *n* (%)	223 (53.2)	458 (54.8)
Prior conventional systemic therapy, *n* (%)	155 (37.0)	318 (38.0)
Prior biologic therapy, *n* (%)	124 (29.6)	254 (30.4)

*Note*. “*n*” values reflect the number of patients who were randomized; the actual number of patients available for each end point may vary.

BID, twice daily; PASI, Psoriasis Area and Severity Index; *SD*, standard deviation; sPGA, static Physician Global Assessment.

**Figure 4:**
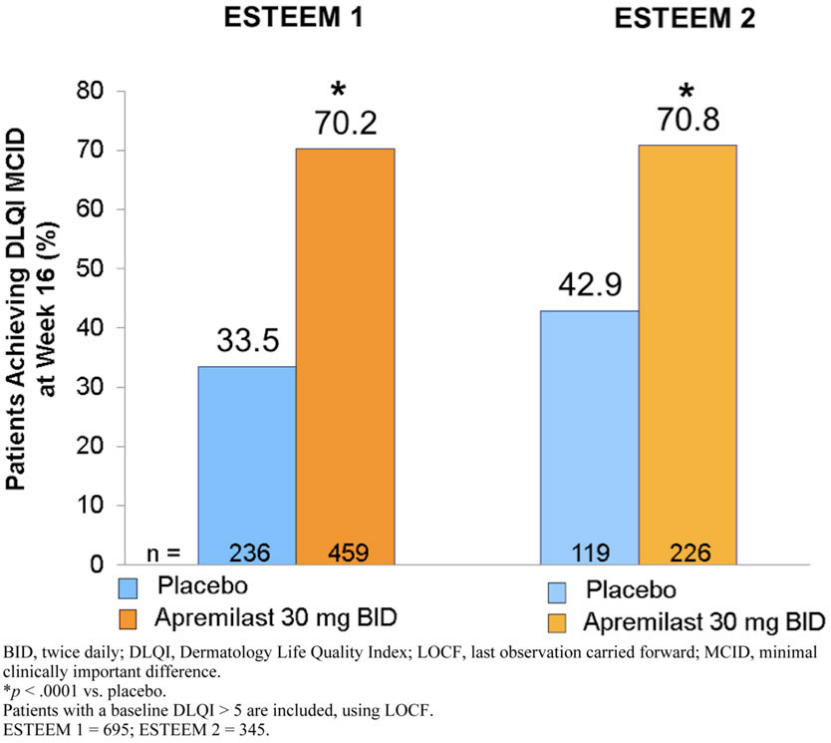
Patients achieving minimal clinically important difference in DLQI score from baseline at week 16 (LOCF).

At week 16, significant improvements in quality of life were observed in patients treated with apremilast versus placebo as measured by Dermatology Life Quality Index (DLQI; Figure 4). Among patients who indicated at baseline that their psoriasis had a significant impact on their quality of life (DLQI score > 5), >70% of patients treated with apremilast reported significant improvement in their quality of life. Significantly, more patients treated with apremilast achieved the minimal clinically important difference (MCID) of a ≥5‐point decrease in DLQI score (indicative of improvement) versus those treated with placebo in both ESTEEM 1 (70.2% vs. 33.5; *p* < .0001) and ESTEEM 2 (70.8% vs. 42.9; *p* < .0001; Papp et al., 2015; Paul et al., 2015).

**Figure 5:**
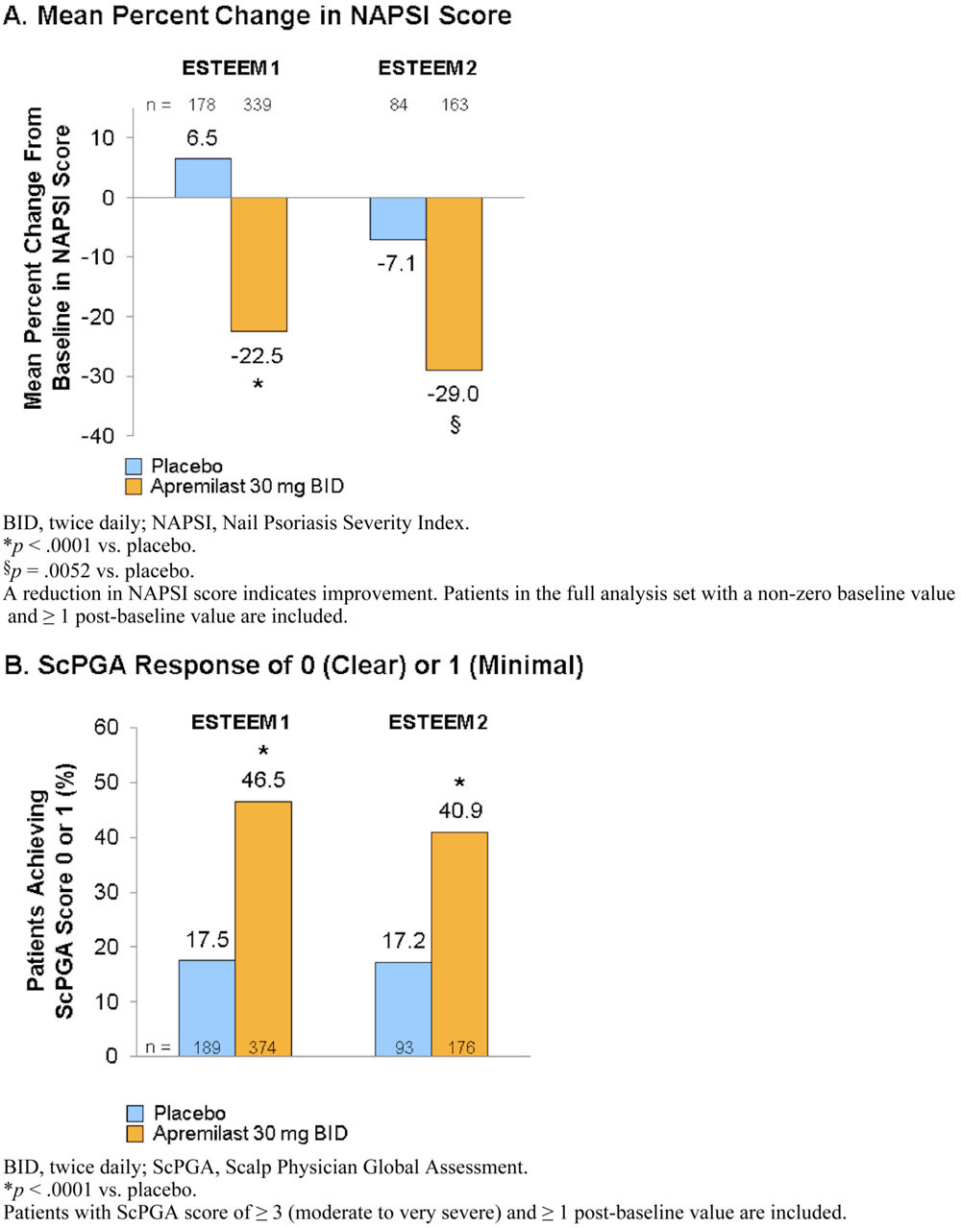
(A) Mean percentage change from baseline in NAPSI score and (B) proportion of patients achieving ScPGA 0 (clear) or 1 (minimal) at week 16.

Patients with nail psoriasis at baseline were assessed using the Nail Psoriasis Severity Index (NAPSI) with improvements (decreases) noted to be significantly greater in patients treated with apremilast versus placebo at week 16 (Figure 5A; Papp et al., 2015; Paul et al., 2015). In the ESTEEM 1 study, mean percent change from baseline in NAPSI score for the target nail (nail that represented the worst nail psoriasis at baseline) in the apremilast group was –22.5% versus +6.5% for the placebo group (*p* = .0001). NAPSI‐50, defined as ≥50% improvement from baseline in NAPSI score, was also greater for patients receiving apremilast versus placebo (33.3% vs. 14.9%) at week 16 (Papp et al., 2015). In the ESTEEM 2 study, mean percent change from baseline in NAPSI score for the target nail in the apremilast group was –29.0% versus –7.1% for the placebo group (*p* = .0052), and NAPSI‐50 achievement was also significantly greater for patients receiving apremilast (44.6%) compared with placebo (18.7%; Paul et al., 2015).

At week 16 among patients with moderate to very severe scalp psoriasis at baseline (Scalp Physician Global Assessment [ScPGA] ≥ 3), significantly more patients receiving apremilast achieved an ScPGA response of 0 (clear) or 1 (minimal) versus placebo in ESTEEM 1 (46.5% vs. 17.5%; *p* = .0001) and ESTEEM 2 (40.9% vs. 17.2%; *p* = .0001; Figure 5B; Papp et al., 2015; Paul et al., 2015).

**Figure 6:**
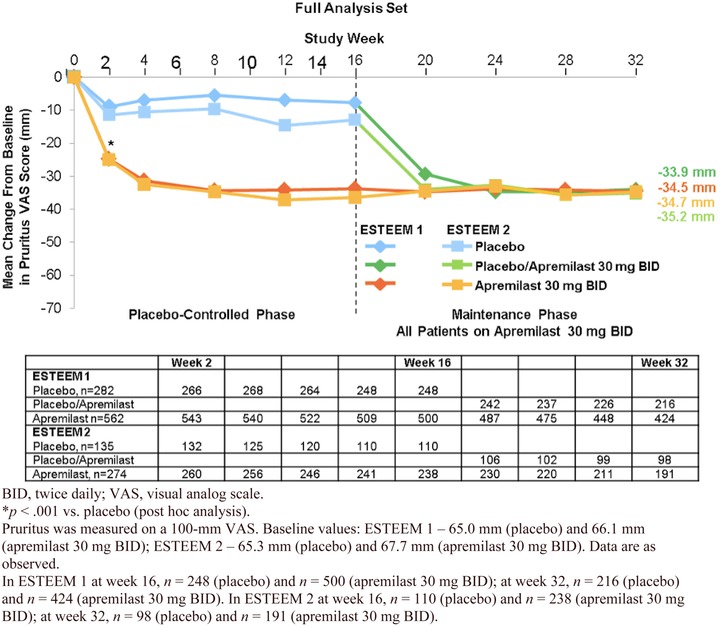
Mean change from baseline in pruritus VAS score (mm) over 32 weeks for ESTEEM 1 and ESTEEM 2.

Additionally, patients’ report of pruritus severity was measured in the ESTEEM program using a 100‐mm visual analog scale (VAS) on which 0 mm corresponded to no itch at all and 100 mm corresponded to the worst itch imaginable. At baseline, mean pruritus VAS scores of 65.0 and 65.3 mm (placebo) and 66.1 and 67.7 mm (apremilast) were observed among the treatment groups in ESTEEM 1 and ESTEEM 2, respectively. At week 16, patients treated with apremilast reported a decrease of nearly 50% in pruritus severity compared with patients receiving placebo (–31.5 mm vs. –7.3 mm in ESTEEM 1; –33.5 mm vs. –12.2 mm in ESTEEM 2; *p* < .0001 for both trials; Figure 6). Improvement in pruritus was observed as early as the first postbaseline visit (week 2) with apremilast, and improvement was maintained through week 32. Patients initially randomized to placebo and switched to apremilast at week 16 exhibited a similar improvement in pruritus at week 32 (Papp et al., 2015; Paul et al., 2015).

**Figure 7:**
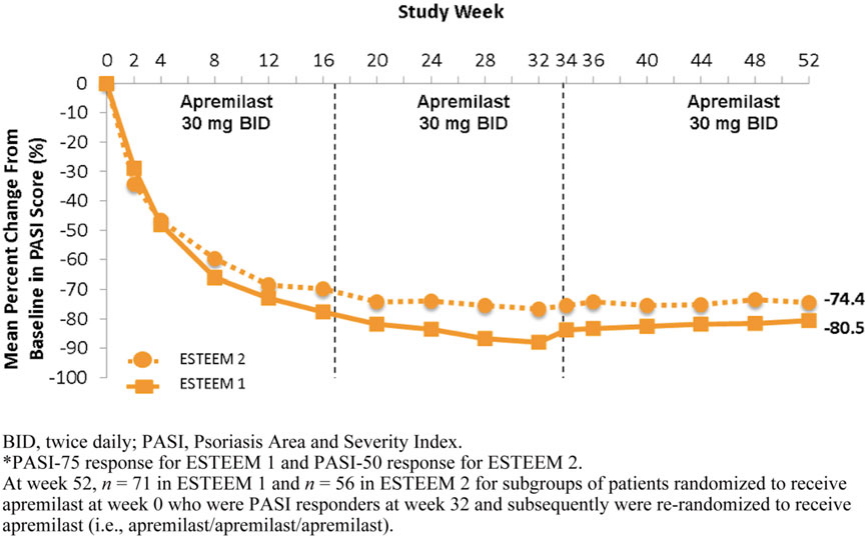
Mean percentage change in PASI over 52 weeks for patients in ESTEEM 1 and ESTEEM 2 who received apremilast from baseline and were PASI responders* at week 32.

During the randomized treatment withdrawal phase (weeks 32‒52), 61.1% of patients who were PASI‐75 responders at week 32 were PASI‐75 responders at week 52 (Figure 7; Papp et al., 2015; Paul et al., 2015). Improvement from baseline in PASI score was maintained among patients who received apremilast at week 0 and were PASI responders (ESTEEM 1: PASI‐75; ESTEEM 2: PASI‐50) at weeks 32–52. For this population in the ESTEEM 1 trial, the mean percent change in the PASI score from baseline was ‒88% at week 32 and ‒81% at week 52 (Papp et al., 2015); likewise, in ESTEEM 2 the mean percent change was ‒77% and ‒74% at weeks 32 and 52, respectively (Paul et al., 2015).

### Clinical safety

The overall safety and tolerability of apremilast in ESTEEM 1 and ESTEEM 2 were reported by pooling the results from both studies. An overview of the adverse events (AEs) is provided in Table 3 (Reich, Papp et al., 2015). Across Period A (weeks 0–16), 57.2% of patients receiving placebo and 68.9% of patients receiving apremilast reported ≥ 1 AE. Most AEs were mild or moderate in severity, and discontinuation rates were low (placebo: 3.8%; apremilast: 5.4%). The incidence of serious AEs was low in both groups (placebo: 2.6%; apremilast: 2.0%; Reich, Papp et al., 2015). The most common AEs (≥ 5% of patients) were diarrhea, nausea, upper respiratory tract infection, nasopharyngitis, tension headache, and headache (Reich, Papp et al., 2015). Most incidences of diarrhea and nausea reported by apremilast‐treated patients were mild in severity and most often occurred in the initial 2 weeks of dosing, and most resolved within 1 month with continued dosing (Reich, Papp et al., 2015). Table 1 shows the recommended initial dosage titration of medication for the first 5 days. The recommended dose of 30 mg BID is started on day 6. The dose‐titration schedule is recommended to reduce gastrointestinal symptoms most commonly seen with treatment initiation.

**Table 3:** Overview of AEs and the most common AEs (≥5% in any treatment group) during the placebo‐controlled period (weeks 0‒16) and the apremilast exposure period (weeks 0 to ≥ 52): Pooled analysis

	Placebo‐controlled period: weeks 0‒16^a^				Apremilast exposure period: weeks 0 to ≥ 52^b^	
	Placebo BID (*n* = 418)	EAIR/100 patient‐years	Apremilast (30 mg) BID (*n* = 832)	EAIR/100 patient‐years	Apremilast (30 mg) BID (*n* = 1184)	EAIR/100 patient‐years
Overview: *n* (%)						
≥1 AE	239 (57.2)	350.3	573 (68.9)	536.4	953 (80.5)	287.4
≥1 Severe AE	15 (3.6)	13.0	32 (3.8)	13.7	97 (8.2)	8.9
≥1 Serious AE	11 (2.6)	9.5	17 (2.0)	7.2	68 (5.7)	6.2
AE leading to drug withdrawal	16 (3.8)	13.8	45 (5.4)	19.2	99 (8.4)	8.8
AE leading to death	1 (0.2)^c^	0.9	1 (0.1)^d^	0.4	2 (0.2)^d^, ^e^	0.2
Reported by ≥5%of patients in anytreatment group: *n* (%)						
Diarrhea	28 (6.7)	25.5	148 (17.8)	74.2	208 (17.6)	22.1
Nausea	28 (6.7)	25.3	138 (16.6)	68.2	188 (15.9)	19.6
Upper respiratory tract infection	27 (6.5)	23.9	70 (8.4)	30.9	200 (16.9)	20.7
Nasopharyngitis	29 (6.9)	25.9	61 (7.3)	26.8	178 (15.0)	17.8
Tension headache	14 (3.3)	12.4	61 (7.3)	27.5	109 (9.2)	10.7
Headache	14 (3.3)	12.4	48 (5.8)	21.2	76 (6.4)	7.1

AE, adverse event; BID, twice daily; EAIR, exposure‐adjusted incidence rate.

a Placebo‐controlled period: Data for weeks 0–16 for patients as initially treated at week 0.

b Apremilast exposure period (weeks 0 to ≥ 52): Data from the first dose of apremilast to the safety cutoff. For patients who received placebo in the treatment withdrawal phase, data for ≤28 days after withdrawal of apremilast are included.

c Completed suicide: In placebo group only.

d The final autopsy report revealed “diffuse lung congestion and bilateral edema, consistent with acute cardiac failure in association with likely sleep apnea and morbid obesity.”

e Cerebrovascular accident. (The patient had a history of diabetes mellitus, hypertension, and hyperlipidemia.)

The exposure‐adjusted incidence rate per 100 patient‐years is defined as 100 × the number of patients reporting the event, divided by patient‐years (up to the first event start date for patients reporting the event).

During the apremilast‐exposure period (weeks 0 to ≥ 52), which included all patients who received apremilast regardless of when it was initiated, similar safety findings were reported. The rate of AEs did not increase over time based on the exposure‐adjusted incident rate, and no new significant AEs emerged with continued exposure (Table 3; Reich, Papp et al., 2015).

Changes in marked laboratory abnormalities were generally infrequent, transient, and comparable between treatment groups, thus no laboratory monitoring is required according to the package insert and FDA rulings (Reich, Papp et al., 2015). Exposure‐adjusted incidence rates of major adverse cardiac events, potential major adverse cardiac events, malignancies, and serious infections (including opportunistic infections) were similar between the placebo and apremilast groups (Reich, Papp et al., 2015). No cases of tuberculosis reactivation were reported during these studies (Papp et al., 2015; Paul et al., 2015). There is no indication that routine laboratory monitoring is necessary during apremilast treatment and is not required in approved labeling (Celgene Corporation, 2015).

In both ESTEEM trials, body weight was measured at selected visits throughout the studies and analyzed by absolute changes and percent change in weight between visits. In the pooled analysis, during Period A (weeks 0‒16) 13.7% of patients who received apremilast versus 5.5% of patients who received placebo experienced weight loss > 5% (Reich, Sobell et al., 2015). During the apremilast exposure period (weeks 0 to ≥ 52), 19.2% of patients experienced a weight decrease of >5%. At week 52, the mean weight change from baseline was –1.99 kg. No association between weight loss and diarrhea or nausea/vomiting was identified; weight loss was not associated with any overt medical sequelae (Reich, Sobell et al., 2015). The incidence of weight loss reported as an AE was low and was reported in 1.4% of patients treated with apremilast 30 mg BID (0 to ≥ 52 weeks); only 2 (0.2%) patients treated with apremilast discontinued because of weight loss (Reich, Sobell, Day, Stevens, & Shah, 2015).

In the ESTEEM 1 and ESTEEM 2 studies, patient‐reported incidences of psychiatric disorders including depression and suicidal ideation were evaluated because depression is a common comorbidity in the psoriasis population (Kurd, Troxel, Crits‐Christoph, & Gelfand, 2010). In the pooled analysis during weeks 0–16, the number of reports of new onset or worsening of depression or depressed mood was low; the percentage of depression AEs was 1.2% in patients receiving apremilast compared with placebo (0.5%). Most AEs of depression were mild or moderate in severity. During the placebo‐controlled period (weeks 0‒16), there was one completed suicide in a patient receiving placebo and one suicide attempt in a patient receiving apremilast. Based on exposure‐adjusted incidence rates per 100 patient‐years, there was no evidence of increasing incidence of depression or suicidal ideation and behavior with longer apremilast treatment (Reich, Papp et al., 2015). As a precaution, clinicians should use apremilast carefully in patients with history of depression and/or suicidal thoughts or behavior and remain alert for the emergence or worsening of depression, suicidal thoughts, or other mood changes in all patients (Celgene Corporation, 2015).

## Clinical perspectives

The American Academy of Dermatology last published guidelines for care of patients with plaque psoriasis and psoriatic arthritis in 2008 (Menter et al., 2008). These guidelines outline the specific role of biologic agents and other conventional treatments for psoriatic disease. Biologic therapies introduced over the last 15 years improved psoriasis treatment tremendously, but associated drawbacks such as safety concerns, loss of efficacy over time, and potential for contraindication emphasize the need for safe, effective, systemic, nonbiologic alternatives. Apremilast provides a unique, nonbiologic, treatment option for adult psoriasis patients with stable moderate to severe plaque type disease or psoriatic arthritis. The National Psoriasis Foundation recently released an updated guidance for psoriasis treatment algorithms and management options, which includes recently approved psoriasis treatments such as apremilast. Patients with psoriasis who may not need or want a biologic or traditional systemic agent—or who prefer the oral route of administration—would be good candidates. Although self‐ or office‐administered biologics may have superior efficacy in some patients, apremilast is a good treatment option because of its favorable safety profile, especially when biologics are contraindicated. In addition, apremilast is dual indicated for plaque psoriasis and psoriatic arthritis, and patients experiencing both might be good candidates for the novel therapy. Table 4 provides a sample checklist with guidelines for assessing candidates starting on apremilast. Laboratory monitoring is not required during apremilast treatment; clinicians should use their own judgment in determining, on a case‐by‐case basis, if tuberculosis testing or laboratory assessments are appropriate.

**Table 4:** Guidelines for assessing patients receiving apremilast

Sample checklist
Assess adherence to prescribed therapy (finished titration pack, any missed doses, challenges getting or remembering to take medication)
Assess for signs and symptoms of depression^a^ (change in eating/sleeping habits, loss of interest in usual activities, poor hygiene, inability to maintain eye contact)
Assess psoriasis severity and improvement at each office visit (BSA: patient's palm = 1% BSA); available tools you may find helpful: Koo‒Menter Psoriasis Instrument or similar tool
Weight measurement (some individuals lose weight during apremilast therapy)
Itch: Scale of 0 (no itch) to 10 (severe pruritus)
Psoriatic arthritis (joint swelling, pain, range of motion [ability to perform activities of daily living]; any nail involvement [usually indicative of PsA])
Potential GI side effects^b^ (loose stools: loperamide if not contraindicated)
Laboratory monitoring if deemed appropriate by the clinician (not mandated by FDA, although may be appropriate based upon presenting comorbid conditions)

AE, adverse event; BSA, body surface area; GI, gastrointestinal; PsA, psoriatic arthritis.

a Screening for depression is not specifically recommended in the package label.

b Diarrhea with apremilast was reported by <20% of patients and was predominantly mild in severity. The highest incidence occurred in the first 2 weeks of treatment and resolved after the first month of dosing.

The likelihood of primary care nurse practitioners treating patients with psoriasis is great given the prevalence of this disease. Many providers of dermatology and rheumatology care are increasingly seeking primary care experts who understand the comorbidities and complexities of psoriatic disease to reduce undertreatment of psoriatic disease symptoms and improve collaborative patient care (Lebwohl et al., 2016). Nurse practitioners may be able to spend more time with patients to identify treatment goals that balance disease severity, comorbidities, and safety concerns with patient quality of life and treatment preferences (Aldredge & Young, 2016). Having comprehensive knowledge of therapeutic options helps create a collaborative and holistic approach to patient care.

## Conclusions

Nurse practitioners should embrace their unique position to partner with patients to ensure comprehensive care is consistently available for all individuals suffering with psoriasis. Patients’ needs are best met when their entire clinical team is knowledgeable regarding all therapeutic options and is vested in providing compassionate care that optimizes outcomes contributing to patients’ overall quality of life. Apremilast is a new therapeutic option for the treatment of psoriatic disease. The results of the ESTEEM clinical trials demonstrate that apremilast significantly reduces the severity of moderate to severe plaque psoriasis, has an acceptable safety profile, and is generally well tolerated. Phase 3 clinical trials also demonstrate that apremilast is an effective and safe treatment option for adult patients with psoriatic arthritis (Kavanaugh et al., 2015). Patients living with psoriasis who are candidates for this orally administered nonbiologic therapy should be educated about the benefits of apremilast. A proactive approach in selecting and preparing patients for an appropriate therapy helps to achieve favorable outcomes. Because of the chronic nature of the disease, a therapeutic relationship between a knowledgeable provider and an informed patient is paramount to successfully managing all aspects of psoriatic disease.
